# Supplementary material for: Comparison of Yamuna (India) and Mississippi River (United States of America) bacterial communities reveals greater diversity below the Yamunotri Glacier
Source: PLoS One. 2024 Jul 5;19(7):e0304664. doi: 10.1371/journal.pone.0304664 (PMC11226128; doi:10.1371/journal.pone.0304664)
Supplement: S1 File — (PDF) [file pone.0304664.s001.pdf]

Request for Permission to Publish Content under CC BY 4.0 License

Dear Rights Holder or Representative,

I have submitted a paper for publication in a journal by PLOS, the Public Library of Science (https://plos.org/). I am hereby requesting permission to use content under your or your organization's management within this paper.

PLOS journals are published under a Creative Commons Attribution 4.0 International license (CC BY 4.0), which allows for unrestricted distribution as well as non-commercial and commercial use of all PLOS journal articles and content, as long as attribution is given to the creator or rights holder of the content. See the full CC BY 4.0 license terms here: https://creativecommons.org/licenses/by/4.0/legalcode.

By granting permission to use this content within a PLOS article, you agree to allow the content's publication under a CC BY 4.0 license without restriction.

To grant me permission to use the content in my PLOS paper, please enter your name, today's date, and your signature on the second page of this form and return the completed form to me at my email address. You may either use an electronic signature or print, sign, and scan or photograph the form.

Thank you very much for your consideration of this request.

Author Name: Osvaldo Martinez

Author Email Address: omartinez@winona.edu

Description of the content which I am seeking permission to use: Fig 1 Maps of the sampling sites on the rivers

Link to the Content: see below

* * *

On behalf of myself or the rights holder, I hereby grant the permission sought herein.

Copyright Holder Name and Title:

Valerie Kellogg ; Designer; prausev@gmail.com

Date:

5/6/24

Signature of Party Granting Permission:

Valerie Kellogg
Designer
Val Kellogg Visuals
665 Oakcrest Dr.
Wadsworth, OH 44281
prausev@gmail.com
4/9/2024

Subject: Permission to Publish Figures under CC BY 4.0 License

To Whom It May Concern,

I am writing to grant permission for the publication of the figures referenced in your communication under the CC BY 4.0 license. I acknowledge that these figures are subject to copyright, and I hereby authorize their use in accordance with the terms of the Creative Commons Attribution 4.0 International License.

Please find attached the figures for which you seek permission. By providing this written permission, I affirm that I am the copyright holder or am duly authorized to grant such permissions on behalf of the copyright holder.

Should you require any further information or clarification regarding this permission, please do not hesitate to contact me at the above-listed contact details.

Thank you for your attention to this matter.

Sincerely,

Valerie Kellogg

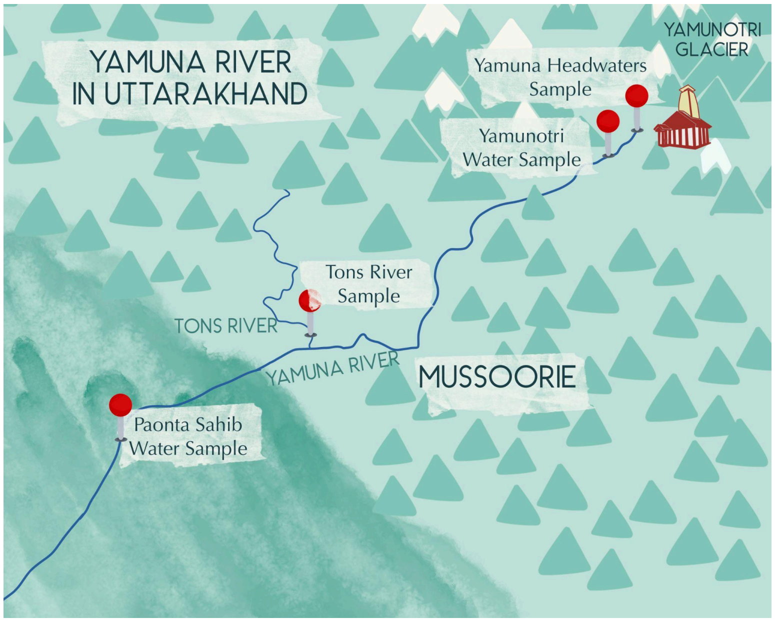
YAMUNA RIVER
IN UTTARAKHAND
YAMUNOTRI
GLACIER
Yamuna Headwaters
Sample
Yamunotri
Water Sample
Tons River
Sample
TONS RIVER
YAMUNA RIVER
MUSSOORIE
Paonta Sahib
Water Sample

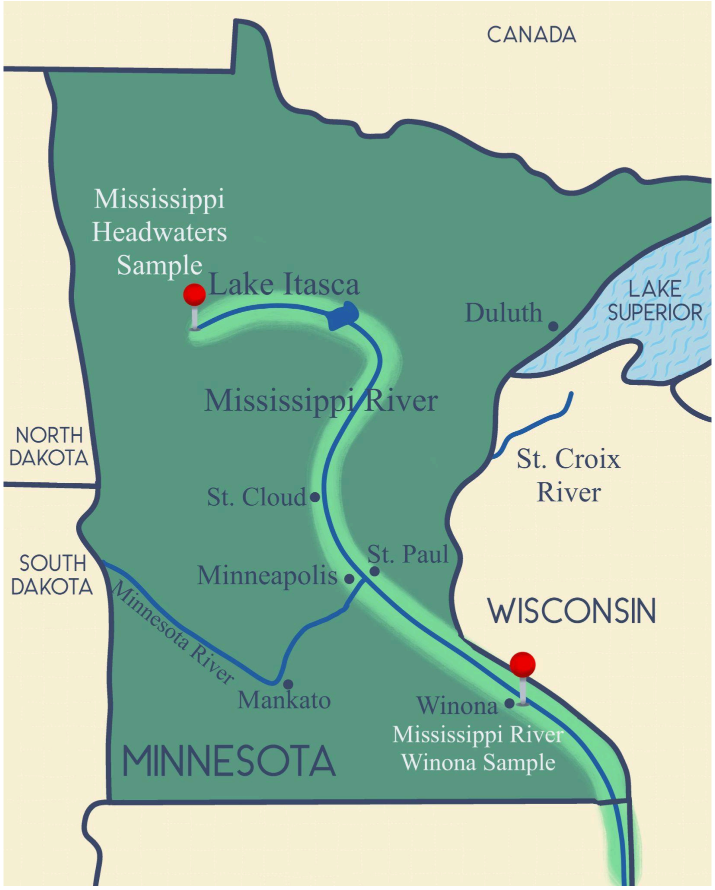
CANADA
Mississippi
Headwaters
Sample
Lake Itasca
Duluth
LAKE
SUPERIOR
Mississippi River
NORTH
DAKOTA
St. Croix
River
St. Cloud
SOUTH
DAKOTA
St. Paul
Minneapolis
WISCONSIN
Minnesota River
Mankato
Winona
Mississippi River
Winona Sample
MINNESOTA

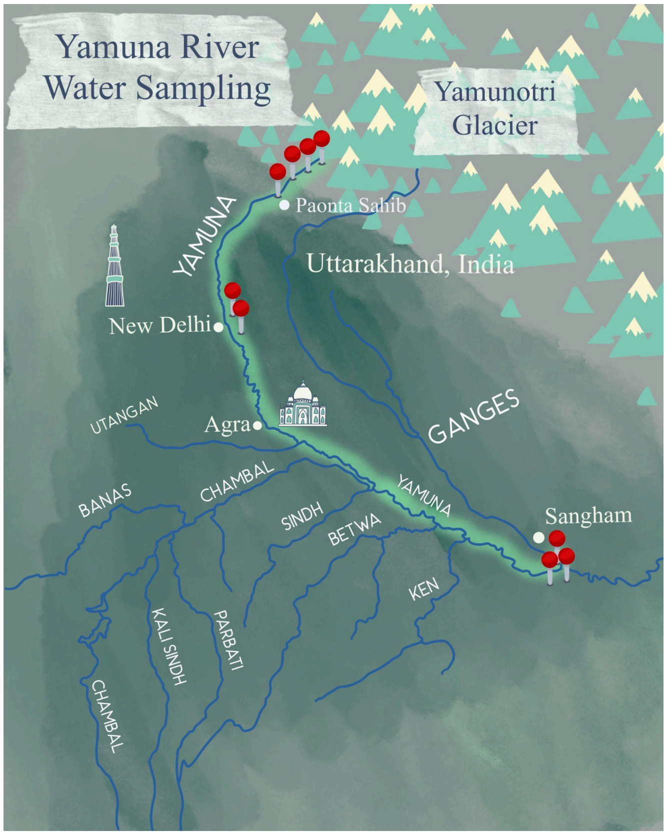
Yamuna River
Water Sampling
Yamunotri
Glacier
YAMUNA
Paonta Sahib
Uttarakhand, India
New Delhi
UTANGAN
Agra
GANGES
BANAS
CHAMBAL
SINDH
YAMUNA
BETWA
Sangham
KEN
KALI SINDH
PARBATI
CHAMBAL
